# Payer mix shifts and profitability at critical access hospitals, 2011 to 2023

**DOI:** 10.1093/haschl/qxag083

**Published:** 2026-04-07

**Authors:** Yusheng Jia, Yue Li, Xueya Cai, Samuel J Enumah

**Affiliations:** Surgical Health Outcomes Research Enterprise, Department of Surgery, University of Rochester Medical Center, Rochester, NY 14642, United States; Department of Environmental Medicine and Public Health Sciences, University of Rochester Medical Center, Rochester, NY 14642, United States; Surgical Health Outcomes Research Enterprise, Department of Surgery, University of Rochester Medical Center, Rochester, NY 14642, United States; Department of Environmental Medicine and Public Health Sciences, University of Rochester Medical Center, Rochester, NY 14642, United States; Surgical Health Outcomes Research Enterprise, Department of Surgery, University of Rochester Medical Center, Rochester, NY 14642, United States; Department of Biostatistics and Computational Biology, University of Rochester Medical Center, Rochester, NY 14642, United States; Surgical Health Outcomes Research Enterprise, Department of Surgery, University of Rochester Medical Center, Rochester, NY 14642, United States; Department of Environmental Medicine and Public Health Sciences, University of Rochester Medical Center, Rochester, NY 14642, United States; Department of Surgery, University of Rochester Medical Center, Rochester, NY 14642, United States

**Keywords:** critical access hospital, financial performance, Medicare, Medicaid, rural health

## Abstract

**Introduction:**

Critical Access Hospitals (CAHs) are essential providers in rural communities but face persistent financial challenges due to narrow operating margins and dependence on public payers. This study examines how changes in payer mix affect both overall and payer-specific profit margins in CAHs from 2011 to 2023.

**Methods:**

Using data from the National Academy for State Health Policy Hospital Cost Tool and the American Hospital Association Annual Survey, we analyzed 15 819 hospital-year observations from 1384 CAHs. We estimated multivariable linear mixed-effects models with profit margin as the dependent variable and 4 payer categories (Commercial, Medicare, Medicaid, and Uncompensated Care) as independent variables. Regression models included fixed year and state effects.

**Results:**

Each percentage point increase in Medicare and Medicaid payer mix relative to Commercial payer mix was associated with a 0.10% and 0.09% point increase in overall hospital operating margin. Higher Medicare, Medicaid, and Uncompensated Care payer mixes corresponded to higher Commercial profit margins.

**Conclusion:**

Hospitals with high public-payer dependence may need high margins on their Commercial cases to offset losses from public payers. Policy efforts to sustain Medicaid coverage and preserve Medicare's cost-based payments are essential to the financial viability of critical access hospitals.

## Introduction

Critical Access Hospitals (CAHs) play a vital role in preserving healthcare access in rural communities across the United States.^1^ Today, CAHs represent more than 1300 facilities across 45 states. Many CAHs offer a range of specialized services, including same-day surgery, infusion therapy, and intensive care.^2,3^ However, CAHs face unique and persistent financial challenges due to narrow operating margins and a reliance on auxiliary funding.^4-6^ Despite the external financial support, CAHs face declining financial margins and rising cost inefficiency.^7,8^ This divergence underscores the precarious balance CAHs maintain between operations and external support.

A central determinant of hospital financial health is payer mix—the distribution of patients by health insurance coverage (eg, Commercial, Medicare, Medicaid). Rural hospitals maintain higher shares of Medicare patients and lower shares of privately-insured patients compared to urban hospitals.^9^ Commercial insurers often provide higher reimbursement rates than public payers for the same services.^10^ Unlike prospective payment system (PPS) hospitals, CAHs are reimbursed by Medicare on a cost basis, which cushions very low volumes but leaves margins sensitive to Medicaid and Commercial rates and to service-line mix (eg, swing beds paid under distinct rules).^11^ Broader policy developments, such as the Patient Protection and Affordable Care Act (ACA), appear to have alleviated some of the financial pressures for hospitals in states that expanded Medicaid coverage. Medicaid expansion improved provider operating margins and financial stability, particularly among rural and small hospitals.^12^ Additionally, hospitals in Medicaid expansion states incurred significantly lower uncompensated care costs (2.6% vs 6.3% of total costs) as compared to hospitals in non-expansion states, and this difference was especially pronounced among rural providers.^13,14^ Despite these insights, CAHs remain understudied in the literature. While Medicaid expansion increased the Medicaid payer mix among hospitalized populations,^15^ the specific dynamics within CAHs, including the Commercial and Medicare payer mix trends and profit margin effects, remain largely unexamined.

Understanding CAH financial dynamics has become increasingly urgent given evolving public policy. The recently passed federal legislation (H.R.1) will substantially alter Medicaid financing through provisions affecting work requirements, provider taxes, and supplemental payments.^16^ Estimates indicate that H.R.1 will decrease Medicaid spending by more than $900 billion, and the uninsured population will increase by 7.5 million.^17,18^ These potential changes make empirical evidence on CAH finances particularly policy relevant. The degree of CAH dependence on public payers and the financial implications of payer mix shifts at CAHs merit further exploration to quantify the degree of hospital financial vulnerability.

This study addresses the knowledge gaps surrounding CAH financial performance by examining how changes in payer mix have affected both overall hospital operating margins and payer-specific operating margins in CAHs. This study describes the trends in Commercial, Medicare, Medicaid, and Uncompensated Care payer mix at CAHs from 2011 to 2023 and examines whether hospital financial performance deteriorates or improves alongside payer mix changes. We hypothesized that CAHs increased their share of Commercial payer mix over time. Furthermore, we hypothesized that shifting payer mix toward higher proportions of public payers (Medicare and Medicaid) would be associated with lower overall operating margins. We further hypothesized that higher Uncompensated Care payer mix would be associated with lower overall operating margins.

## Study data and methods

### Data sources and sample

We conducted a longitudinal analysis using 2 primary data sources. Financial data on hospital margins and payer mix were obtained from the National Academy for State Health Policy (NASHP) Hospital Cost Tool, which includes annual Medicare Cost Report data for approximately 4600 US hospitals. Key measures in NASHP include overall operating margins and payer-specific margins (for Commercial, Medicare, and Medicaid payers) from 2011 to 2023.^19^ NASHP provides detailed financial data including revenues, costs, and profitability for hospitals nationwide, and it has been used in previous literature on hospital financing and management.^6,20^ Using the Medicare Provider Identification number, we linked the NASHP data to hospital characteristics and operating information from the American Hospital Association (AHA) Annual Survey.^21^ The AHA Annual Survey collects information on hospital services, utilization, expenses, and staffing patterns, and has been used in a variety of hospital and health services research studies.^22-25^

We analyzed US hospitals that maintained the CAH designation during the study period of 2011-2023 and that reported the necessary data in the NASHP and AHA sources. For a more complete description of the dataset construction, see the Appendix.

### Measures

The primary outcomes were operating profit margin and payer-specific profit margins. We defined the operating profit margin as operating profit (or loss) divided by net patient revenue. This is a common financial measure to characterize organizational profitability and represents earnings based on hospital patient services.^26-28^ We defined payer-specific profit margins for Commercial, Medicare Fee-for-Service (FFS), and Medicaid. Payer-specific margins were calculated as the profit attributable to each payer group divided by that group's net patient revenue, reflecting whether payments exceeded, met, or fell short of the costs of care. Given the known skewness in hospital profit margin distributions, we addressed outliers by excluding observations below the 1st percentile and above the 99th percentile of operating profit margin.

The key independent variables included 4 mutually exclusive hospital payer proportions, measured as the percentage of hospital services (by charges) attributable to each major payer category: Commercial, Medicare, Medicaid, and Uncompensated Care. NASHP's definition of “Commercial” payer mix included non-Medicare and non-Medicaid sources such as employer-sponsored plans, Tricare, and the Veteran Affairs (VA) insurance. Medicare payer mix included both Medicare FFS and Medicare Advantage. Medicaid payer mix included fee-for-service and managed care, State Children's Health Insurance Program (SCHIP), and other low-income government programs. Uncompensated Care payer mix represented a combined category including charity care, uninsured patients, and bad debt, according to NASHP. Higher values represent greater financial reliance on that payer group.

Additional hospital characteristics included Core-based statistical area (CBSA), teaching status, bed size, ownership (government-owned, nonprofit, or for-profit), health system membership status, market concentration, annual surgical operations, and inpatient occupancy. We defined each unique healthcare market according to the Dartmouth Atlas Hospital Referral Region (HRR), and we determined the level of market concentration using the Herfindahl–Hirschman Index (HHI).^29,30^ We extracted adjusted patient discharge volume from NASHP (see Appendix). We calculated the sum of the squared share of adjusted patient discharges for each hospital in the competing HRR in each year. We defined high-volume hospitals as those facilities that reported a surgical volume at or above the 75th percentile for a given year, and the remaining hospitals were categorized as low volume.

### Statistical analysis

We first conducted descriptive analyses, including baseline facility characteristics and crude trends in payer mix and profit margins over time. The median and the interquartile range (IQR) were reported for continuous variables, and absolute and relative frequencies were presented for categorical variables. Trends were plotted using annual means to visually examine changes in payer composition and financial performance.

We used linear mixed-effects models to examine the association between hospital payer mix and profit margins (see the Appendix for full model details). We used hospital-level random intercepts to control for within-hospital clustering effect, and we included year and state fixed effects to account for time and state level heterogeneity. Separate models were estimated for operating, Commercial, Medicare, and Medicaid profit margins (in percentage points). We included the Medicare, Medicaid, and Uncompensated Care payer shares simultaneously, while omitting the Commercial payer mix (the reference category). Under this specification, the regression coefficients represent the estimated change in profit margin associated with a one-percentage-point shift from the Commercial payer mix to the specified payer category.

In sensitivity analyses, we assessed robustness using an alternative cutoff (winsorization at 1st/99th percentile instead of trimming), a balanced panel analysis restricted to hospitals that reported data in each year, and adding Medicaid expansion status as a covariate. All hypothesis tests were two-sided, with a significance threshold of *P* < 0.05 for coefficient estimates. Analyses were performed using Stata/MP 19.5.

### Limitations

Our analysis has several limitations. First, although we used comprehensive national data from 2011 to 2023, our analysis is observational and cannot establish causality. Payer mix is not randomly assigned but reflects geographic location, community demographics, and hospital strategic decisions. While we adjusted for hospital characteristics, market factors, and year and state fixed effects, unmeasured confounding (eg, local policy changes and negotiated private payment rates) may have impacted our findings. Additionally, our panel is unbalanced due to data reporting gaps and rural hospital closures.^31^ Despite determining that the primary findings were robust between balanced and unbalanced panels, there may be potential confounding from strategic decisions that might influence which CAHs remain in the sample. Within the unbalanced panel, hospitals may have closed or changed ownership, which may have changed their frequency or ability to report data.

Second, while we examined associations between payer mix and margins, we did not capture other revenue sources, such as disproportionate share hospital (DSH) payments or other policy-driven subsidies. The DSH payments represent government subsidies that are not included in the operating margin, given that they are not directly related to patient care. Hospitals may receive additional payments that are not reflected in their operating margins, which could mediate observed relationships.^32^

Third, the lack of granularity of specific payers within this dataset may limit these findings. The NASHP Cost Tool combines employer self-funded plans, Tricare, and the Veterans Affairs (VA) into a single category of “Commercial.” Tricare and the VA represent military-related health insurance systems that are not traditionally considered “Commercial.” Given that the Tricare program covers approximately 9.5 million beneficiaries,^33,34^ the VA system covers approximately 9.2 million beneficiaries,^35^ and employer-sponsored plans represent approximately 165 million people,^36^ the impact on our outcomes of combining the Tricare and VA charges into the “Commercial” payer mix may be limited. Nevertheless, we are unable to accurately estimate the specific distribution of these payers within this dataset. Additionally, the cost-to-charge ratio methodology used to derive payer-specific margins in NASHP assumes proportional cost distribution across payers, which may introduce measurement error if payer populations differ in resource intensity.

Fourth, the analysis incorporates comprehensive payer mix categories that differ in composition from the profit margin groups for Medicaid and Medicare. We sought to explore factors associated with the Medicaid-only profit margin, but we chose to combine Medicaid, SCHIP, and low-income government programs as a payer mix. The Medicare profit margin only represents traditional Medicare. Our analyses did not include Medicare Advantage (MA) specific profit margins, given data limitations within the NASHP dataset for CAHs. Given the notable rise in MA enrollment over the past decade,^37^ future studies are needed to investigate MA-specific profit margins.

Fifth, we did not adjust significance thresholds for multiple comparisons across our 4 outcome models, as each model addresses a conceptually distinct dimension of CAH financial performance (overall operating margin and payer-specific margins). Our findings should be interpreted as hypothesis-generating rather than confirmatory, and replication in independent datasets is warranted.

Finally, we focused on hospital-level financial outcomes and did not assess potential downstream effects on patient access, quality of care, or long-term sustainability. Future research should integrate financial performance with clinical and community health outcomes to better inform rural health policy.

## Results

Our analysis included 15 819 hospital-year observations from 1384 CAHs between 2011 and 2023. In 2023, most CAHs in the sample had 25 or more beds (57.6%) and were located in rural areas (61.3%, Table 1). The majority were either non-profit (58.4%) or public/government-owned (38.4%) facilities. About half (50.7%) of the CAHs were part of a health system. In 2023, the median annual surgical volume was 883 operations (IQR [275, 1699]), and the median inpatient occupancy was 28.3% (IQR [17.1%, 43.6%]). In 2023, the payer mix distribution was 44.6% Commercial, 35.0% Medicare, 15.0% Medicaid, SCHIP, and Low-Income Government Programs, and 3.0% Uncompensated Care. The median hospital operating margin was 4.4% (IQR [−5.9%, 14.3%]). Across Commercial, Medicare, and Medicaid payers, the median payer-specific operating margins were 21.5% (IQR [0.5%, 39.3%]), 0.3% (IQR [−0.5%, 1.6%]), and −20.2% (IQR [−55.2%, 5.4%]).

**Table 1:** Characteristics of critical access hospitals, 2011 vs 2023.

	2011	2023
Variables	Summary	Summary
**Number of hospitals**	1098	1242
**CBSA, *n* (%)**		
** Metro**	189 (17.2%)	254 (20.5%)
** Micro**	198 (18.0%)	226 (18.2%)
** Rural**	711 (64.8%)	762 (61.3%)
**Teaching status, *n* (%)**		
** Non-teaching**	1098 (100.0%)	1083 (87.2%)
** Teaching**	0 (0.0%)	159 (12.8%)
**Bed size, *n* (%)**		
** <25**	406 (37.0%)	526 (42.4%)
** ≥25**	692 (63.0%)	716 (57.6%)
**Ownership, *n* (%)**		
** For-profit**	58 (5.3%)	40 (3.2%)
** Governmental**	450 (41.0%)	477 (38.4%)
** Non-profit**	590 (53.7%)	725 (58.4%)
**System member status, *n* (%)**		
** System member**	526 (47.9%)	630 (50.7%)
** Independent**	572 (52.1%)	612 (49.3%)
**Market concentration, *n* (%)**		
** Highly concentrated**	364 (33.1%)	391 (31.5%)
** Moderately concentrated**	208 (19.0%)	270 (21.7%)
** Unconcentrated**	526 (47.9%)	582 (46.8%)
**Total surgical operations, median (IQR)**	617 (185, 1277)	883 (275, 1699)
**Total surgical operations Category, *n* (%)**		
** Low volume**	821 (74.8%)	932 (75.0%)
** High volume**	277 (25.2%)	310 (25.0%)
**Inpatient occupancy (%), median (IQR)**	33.0 (21.5, 47.7)	28.3 (17.1, 43.6)
**Profit margins**		
** Operating profit margin (%),** **median (IQR)**	9.7 (0.03, 19.0)	4.4 (−5.9, 14.3)
** Commercial profit margin (%),** **median (IQR)**	30.6 (11.0, 46.4)	21.5 (0.5, 39.3)
** Medicare profit margin (%),** **median (IQR)**	2.8 (1.2, 5.7)	0.3 (−0.5, 1.6)
** Medicaid profit margin (%),** **median (IQR)**	−19.7 (−56.7, 5.1)	−20.2 (−55.2, 5.4)
**Payer mix**		
** Commercial payer mix (%), median (IQR)**	39.2 (32.5, 47.1)	44.6 (38.0, 51.3)
** Medicare (traditional and Medicare** **Advantage) payer mix (%), median (IQR)**	40.5 (33.7, 47.5)	35.0 (27.4, 42.2)
** Medicaid, SCHIP, and Low-Income** **Government Programs payer mix (%),** **median (IQR)**	11.7 (7.0, 17.3)	15.0 (9.7, 20.9)
** Charity Care, uninsured and bad debt** **payer mix (%), median (IQR)**	5.3 (3.2, 8.7)	3.0 (1.9, 5.1)

Source: Authors' analysis of data from the National Academy for State Health Policy (NASHP) Hospital Cost Tool and the American Hospital Association (AHA) Annual Survey, calendar year 2011 and 2023.

Notes: Values are presented as number and percentage [*n* (%)] for categorical variables, and as medians with interquartile ranges [median (IQR)] for continuous variables. CBSA refers to Core-Based Statistical Area classification (metro, micro, or rural).

**Figure 1:**
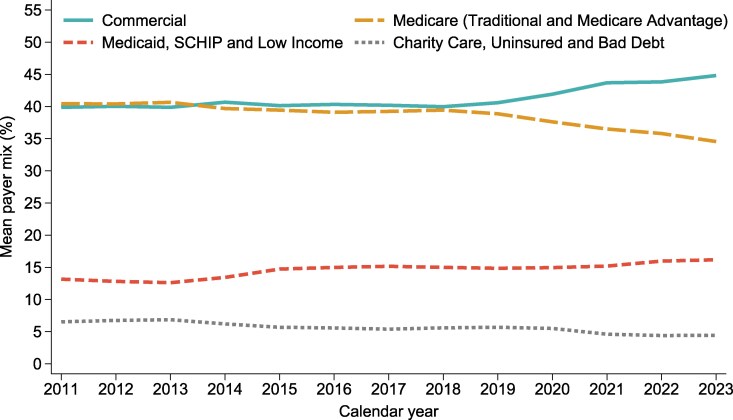
Trends in payer mix among critical access hospitals, 2011-2023. Source: Authors' analysis of data from the National Academy for State Health Policy (NASHP) Hospital Cost Tool and the American Hospital Association (AHA) Annual Survey, calendar year 2011-2023. Notes: This figure shows the mean payer mix in percentage for Commercial insurance, Medicare, Medicaid, and Uncompensated Care across all critical access hospitals from 2011 to 2023 using NASHP data. Commercial payer mix was defined as the percentage of hospital services provided to all other commercial payers (insured plans, employer self-funded plans, Tricare, VA, etc.), as measured by hospital charges. Medicare payer mix was defined as the percentage of total hospital services provided to Medicare patients (including both traditional and Medicare Advantage), as measured by hospital charges. Medicaid payer mix was defined as the percentage of hospital services provided to Medicaid, State Children's Health Insurance Program (SCHIP), and other low-income government programs patients, as measured by hospital charges. Uncompensated Care payer mix was defined as percentage of hospital services provided to charity care, uninsured and bad debt patients, as measured by hospital charges.

CAHs experienced shifts in the average payer mix over the study period (Figure 1). The Medicaid, SCHIP, and Low-Income Government Programs payer mix increased from 13.2% to 16.2%, the Medicare payer mix declined from 40.4% to 34.6%, and the Uncompensated Care payer mix declined from 6.5% to 4.4%. Commercial payer mix remained the largest payer category and increased from 39.9% to 44.8%. In 2023, Medicare and Medicaid collectively accounted for approximately half of the CAHs payer mix.

**Figure 2:**
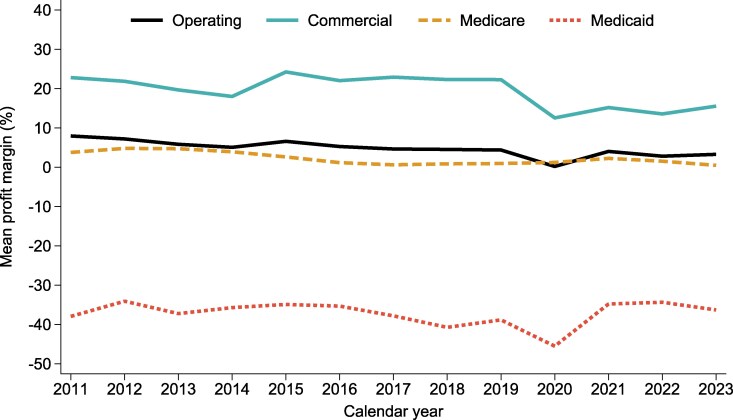
Trends in profit margins by payer type among critical access hospitals, 2011-2023. Source: Authors' analysis of data from the National Academy for State Health Policy (NASHP) Hospital Cost Tool and the American Hospital Association (AHA) Annual Survey, calendar year 2011-2023. Notes: This figure displays average annual profit margins for Critical Access Hospitals (CAHs) from 2011 to 2023 using NASHP data, including overall operating profit margin and margins by payer type: Commercial, Medicare Fee-for-Service (FFS), and Medicaid. Operating profit margin was calculated as Operating Profit (Loss) divided by Net Patient Revenue and reflects earnings from hospital patient services. Payer-specific profit margins were defined as the profit attributed to each payer (Commercial, Medicare, and Medicaid) divided by net patient revenue from that payer.

Despite shifts in the payer mix, the profitability gap between payer types remained persistent over time (Figure 2). CAHs generally maintained positive average operating margins, though averages declined from 8.0% in 2011 to 3.3% in 2023. Commercial patients consistently generated high margins (22.8% in 2011 and 15.6% in 2023). Medicare average profit margins hovered near break-even (from 3.8% in 2021 to 0.5% in 2023). Medicaid average profit margins remained negative throughout the study period (−37.9% in 2011 and −36.3% in 2023).

In our mixed-effects models, different payer mix categories were associated with CAH financial outcomes (Table 2). In the model for overall operating margin, a higher Medicare payer mix was associated with a higher operating margin (coefficient 0.10, 95% confidence interval (CI) [0.05, 0.15], *P* < 0.001). Similarly, a higher Medicaid payer mix was associated with a higher operating margin (coefficient 0.09, 95%CI [0.04, 0.14], *P* < 0.001). In contrast, Uncompensated Care payer mix was associated with a decrease in the overall margin (coefficient −0.18, 95%CI [−0.29, −0.07], *P* = 0.001).

**Table 2:** Effect estimates of payer mix on profit margins among critical access hospitals, 2011-2023.

	(1)	(2)	(3)	(4)
	Operating profit margin (%)	Commercial profit margin (%)	Medicare profit margin (%)	Medicaid profit margin (%)
	Coef. 95% CI *P*-value	Coef. 95% CI *P*-value	Coef. 95% CI *P*-value	Coef. 95% CI *P*-value
Commercial payer mix (%)	Ref.	Ref.	Ref.	Ref.
Medicare FFS and Medicare Advantage payer mix (%)	0.10***	0.44***	0.01**	−0.22**
	[0.05,0.15]	[0.29,0.59]	[0.00,0.03]	[−0.44,−0.00]
	<0.001	<0.001	0.038	0.048
Medicaid, SCHIP, and Low-income payer mix (%)	0.09***	0.97***	0.03***	−0.50***
	[0.04,0.14]	[0.80,1.15]	[0.01,0.04]	[−0.78,−0.22]
	<0.001	<0.001	<0.001	0.001
Charity care, uninsured, and bad debt payer mix (%)	−0.18***	2.16***	−0.01	0.41*
	[−0.29,−0.07]	[1.84,2.47]	[−0.10,0.08]	[−0.02,0.84]
	0.001	<0.001	0.793	0.062

Source: Authors' analysis of data from the National Academy for State Health Policy (NASHP) Hospital Cost Tool and the American Hospital Association (AHA) Annual Survey, calendar year 2011-2023.

Notes: This table presents results from linear mixed-effects regression models estimating the association between hospital payer mix and 4 financial outcomes among Critical Access Hospitals from 2011 to 2023. All models were estimated on a panel of 1384 CAHs with a total of 15 819 hospital-year observations. The dependent variables were profit margins by payers, each expressed as a percentage. All models included hospital-level random intercepts (clustered at the hospital level) and fixed effects for year and state. Key independent variables were the percentage of hospital services (by charges) attributable to Commercial, Medicare, Medicaid, and Uncompensated Care payers. Each cell reports the regression coefficient, followed by the 95% confidence interval in brackets and *P*-value underneath. Robust standard errors were clustered at the hospital level.

For the mixed-effects models examining payer-specific profit margins, we identified that higher Medicare, Medicaid, and Uncompensated Care payer mixes were associated with higher commercial specific profit margins. For each one percent increase in Medicare, Medicaid, and Uncompensated Care payer mix, the Commercial profit margin changed by 0.44 (95%CI [0.29, 0.59], *P* < 0.001), 0.97 (95%CI [0.80, 1.15], *P* < 0.001), and 2.16 (95%CI [1.84, 2.47], *P* < 0.001), respectively. For each one percent increase in Medicare and Medicaid payer mix, the Medicare FFS profit margin increased by 0.01 (95%CI [0.00, 0.03], *P* = 0.038) and 0.03 (95%CI [0.01, 0.04], *P* < 0.001), respectively. For each one percent increase in Medicare and Medicaid payer mix, the Medicaid profit margin decreased by 0.22 (95%CI [−0.44, −0.00], *P* = 0.048) and 0.50 (95%CI [−0.79, −0.23], *P* < 0.001).

High surgical volume was associated with significantly higher operating profit margins (coefficient 1.68, 95%CI [0.93, 2.43], *P* < 0.001) compared to low surgical volume (Table A1 in Appendix). Additionally, CAHs with high surgical volume demonstrated higher Commercial profit margins (coefficient 2.39, 95%CI [0.57, 4.22], *P* = 0.010), lower Medicare profit margins (coefficient −0.27, 95%CI [−0.55, −0.00], *P* = 0.048), and higher Medicaid profit margins (coefficient 5.93, 95%CI [2.33, 9.52], *P* = 0.001) compared to CAHs with low surgical volume.

Our primary findings were generally robust to alternative approaches. Using winsorization at the 1st/99th percentiles, restricting the analysis to a balanced panel, and adding the additional covariate of Medicaid Expansion status yielded similar coefficients and statistical significance (Appendix Tables A2–A4).

## Discussion

This study examined the relationship between payer mix and financial performance among CAHs from 2011 to 2023. Consistent with our hypothesis, commercial payer mix increased over time, and a higher share of uncompensated care was associated with lower overall operating margins. Contrary to our hypothesis, we found that higher Medicare and Medicaid payer mixes were significantly associated with higher, though modest, overall operating profit margins. We also found that CAHs with a higher share of public payers (Medicare and Medicaid) experienced higher Commercial-specific profit margins.

Our study offers notable insight regarding the relationship between public payer mix and overall hospital financial performance. The magnitude of the estimated associations between Medicaid and Medicare payer mixes are small but positive. This result runs counter to the common concern that serving more publicly insured patients automatically undermines hospital profitability. One explanation is that the Affordable Care Act's 2014 Medicaid Expansion positively impacted CAHs efficiency or costs. Previous literature highlights substantial reductions in uncompensated care and increases in Medicaid revenue and hospital profit margins in hospitals within states that expanded Medicaid.^38-40^ Another plausible explanation is that hospitals with greater public payer dependence or uncompensated care need (or have achieved) higher commercial profit margins to survive.

Our analysis suggests that CAHs with more publicly insured patients and higher uncompensated care have higher Commercial profit margins. While our analytic approach is not causal, there may be stronger motivation for cross-subsidization in CAHs when they provide care for a higher proportion of Medicaid, low-income, or uninsured patients. Prior literature suggests that, compared to other hospitals, CAHs tended to have relatively limited capacity for active cost-shifting,^6,41^ given their constrained negotiating power in dealing with private payers. Although this is generally true for CAHs, one potential interpretation is that CAHs with higher public-payer dependence, while relying on the adequacy of public payments themselves, may differ in their approach to Commercial reimbursement or expense management from their remaining Commercial cases. This approach may make up for shortfalls from other low or negative-margin payer mixes. On the other hand, while increased uncompensated care in CAHs was positively associated with Commercial profit margins, it was also associated with a decrease in overall operating margins. These findings suggest that CAHs may not have the ability to fully compensate for the loss due to uncompensated care with increased commercial profits.

Importantly, CAHs maintain important additional mechanisms to limit the negative financial impact of caring for publicly insured patients. First, CAHs receive Medicare cost-based reimbursement at 101% of reasonable costs, and therefore, higher Medicare volume does not inherently reduce overall profit margins.^42^ Second, Medicaid cost-based reimbursement varies across states,^43^ but cost-based reimbursement may also explain the relatively neutral impact of Medicaid payer mix on overall hospital operating margin. Third, the association between operating margin and Medicaid payer mix may reflect the role of coverage in reducing uncompensated care. As previously uninsured patients gained Medicaid coverage, a proportion of otherwise uncompensated care converted to compensated care for hospitals. Taken together, these factors help explain why a heavier public-payer mix may not translate into worse overall margins for CAHs. Lastly, we cannot fully rule out residual confounding, as CAHs with increased public payer mix may differ systematically in operational efficiency or access to supplemental funding.

While our study identified a positive association between Medicaid payer mix and overall operating profit, our results were modest in the magnitude of the relationship. Chatterjee et al. (2021) studied 1158 CAHs in states that did and did not expand Medicaid based on the ACA, and the authors did not identify statistically significant increases in Medicaid inpatient days or in hospital operating margins within expansion states compared to non-expansion states.^44^ Despite operating within the broader healthcare ecosystem, CAHs encounter unique market forces that may create added complexity for financial sustainability. For CAHs, an increase in Medicaid payer mix may provide financial stability by reducing uncompensated care, but the ability to convert that into profitability may hinge on reimbursement adequacy and efficient care delivery for Commercial patients.

Our study also revealed that CAHs with high surgical volume achieved higher operating profit margins compared with CAHs with low surgical volume. This finding is consistent with prior literature on the financial benefits of maintaining robust surgical services in rural settings.^45^ Prior studies have shown that each additional surgical service offered at a CAH is associated with notable gains in hospital income and overall financial performance.^45-47^ These associations are especially relevant considering the similar inpatient capacity across CAHs (typically capped at 25 beds),^48^ suggesting that financial gains may stem from outpatient or short-stay surgical procedures rather than inpatient volume. Given that CAHs are a predominant source of care in rural areas,^49^ surgical services may represent a modifiable pathway to improve financial sustainability for CAHs. Expanding procedural capabilities (eg, same-day surgery) could help stabilize margins without exceeding capacity limits. Nevertheless, policy efforts to expand procedural capabilities at CAHs should consider tradeoffs. For many rural communities, hospitals provide emergency and inpatient care as well as primary care.^50^ Expanding surgical services requires capital investment that may not be feasible for financially distressed hospitals,^51,52^ and excessive focus on profitable procedural services could potentially divert resources from primary care and other essential services. Our findings suggest surgical services represent one potential pathway to financial sustainability, but hospital-specific and community-specific factors should guide such decisions.

Our findings carry implications for potential Medicaid policy changes. As uncompensated care potentially increases from the loss of Medicaid coverage, CAHs may have limited additional ability to offset these losses with Commercial profits. Rural hospitals, already operating on thin margins, may be particularly vulnerable to coverage contractions. Recent analyses have highlighted concerns about the downstream effects of Medicaid financing changes on safety-net providers.^53,54^ Our findings underscore the importance of considering rural hospital financial stability when evaluating policy proposals affecting Medicaid coverage and reimbursement.

## Conclusion

Among CAHs from 2011 to 2023, higher Medicaid and Medicare payer mixes were associated with modest increases in overall operating profit margins, possibly related to higher Commercial profit margins. A shift toward uncompensated care was linked to lower overall operating profit margins. Maintaining adequate reimbursement from public programs and Commercial payers is essential to CAH sustainability. These findings highlight the importance of sustaining Medicaid coverage to support the financial health of rural hospitals and avoid the financial strain of uncompensated care.

## Supplementary Material

qxag083_Supplementary_Data
