# Supplementary material for: Payer mix shifts and profitability at critical access hospitals, 2011 to 2023
Source: Health Aff Sch. 2026 Apr 7;4(4):qxag083. doi: 10.1093/haschl/qxag083 (PMC13101979; doi:10.1093/haschl/qxag083)
Supplement: qxag083_Supplementary_Data [file qxag083_supplementary_data.zip › Appendix Materials_RR3_3_18_26_C.docx]

Supplementary Materials – Appendix

Table of Contents

[Additional Detailed Methodology 2](#_Toc223684089)

[Table A1 Effect Estimates of Payer Mix on Profit Margins Among Critical Access Hospitals, 2011-2023 5](#_Toc223684090)

[Table A2 Sensitivity Analysis – Winsorization at 1st/99th percentiles 7](#_Toc223684091)

[Table A3 Sensitivity Analysis – Balanced Panel 9](#_Toc223684092)

[Table A4 Sensitivity Analysis – Alternative Models with Medicaid Expansion Control 11](#_Toc223684093)

# Additional Detailed Methodology

**Data Sources and Sample**

To conduct our analyses, we constructed our dataset through multiple steps integrating financial performance data with hospital characteristics. Our analysis was based on administrative data from two primary sources: the National Academy for State Health Policy (NASHP) Hospital Cost Tool and the American Hospital Association (AHA) Annual Survey Database.

The NASHP Hospital Cost Tool derives financial measures from Medicare Cost Reports filed annually by hospitals. Payer-specific profit margins are calculated as payer-specific operating profit divided by payer-specific net patient revenue. For example, Commercial Operating Profit Margin equals Commercial Operating Profit divided by Commercial Net Patient Revenue. To allocate hospital operating costs across payer categories, NASHP applies a cost-to-charge ratio methodology: total hospital operating costs eligible for Medicare reimbursement are distributed to each payer proportionally based on each payer's share of hospital charges. This approach assumes that the cost of providing services is proportional to charges across payer groups. The NASHP tool reports separate margins for Commercial payers (including employer-sponsored plans, self-funded plans, and other plans such as TriCare and VA), Medicare FFS, Medicaid, and other categories. Further methodological details are available from the NASHP Hospital Cost Tool documentation (https://tool.nashp.org/).

We matched hospitals between the NASHP and AHA databases using Medicare Provider Numbers (CCN). The CCN was standardized as a 6-digit string format to ensure consistent matching across data sources. We successfully matched hospitals that appeared in both databases using CCN and year, retaining only observations present in both sources.

Our analysis focused on hospitals designated as Critical Access Hospitals (CAHs) that maintained this designation throughout the study period. We excluded special payment designation hospitals, and facilities with incomplete financial reporting.

Adjusted patient discharges is a calculated value that represents both inpatient and outpatient discharge volume. Per NASHP, adjusted patient discharges is calculated by multiplying inpatient discharge volume by hospital charges divided by inpatient hospital charges.

**Final Analysis Dataset Construction**

Of the initial 17,238 CAH-year observations identified in the merged dataset:

- 902 observations (5.2%) were excluded due to missing profit margin data
- 179 observations (1.0%) were excluded due to missing payer mix information for key payers
- 16 observations (0.1%) were due to missing control variables
- 322 observations (1.9%) were excluded as outliers (below 1st percentile or above 99th percentile of operating profit margin values)

The final analysis file contained 15,819 hospital-year observations from 2011 through 2023, representing 1,384 unique Critical Access Hospitals. This corresponds to an average of 1,216 hospitals per year, with yearly observations ranging from 1,098 to 1,255 hospitals. The unbalanced panel structure reflects temporary data reporting gaps and the exclusion of observations with incomplete financial metrics.

**Statistical Analysis**

We employed linear mixed-effects models to examine the association between payer mix and financial performance, accounting for the hierarchical structure of our data (repeated observations nested within hospitals). The general specification was:

$$ProfitMargin_{it}=\alpha+\beta_{1}{MedicareMix}_{it}+\beta_{2}{MedicaidMix}_{it}+\beta_{3}{UncompensatedMix}_{it}+\gamma X_{it}+u_{i}+\lambda_{t}+\delta_{s}+\epsilon_{it}$$

Where:

- i indexes hospitals and t indexes years
- ProfitMargin represents one of four outcomes: overall operating margin, Commercial operating margin, Medicare operating margin, or Medicaid operating margin
- MedicareMix, MedicaidMix, and UncompensatedMix variables represent the percentage of hospital services provided to each patient group, as measured by hospital charges; CommercialMix variable (i.e. Commercial payer mix) was omitted as the reference group
- X represents hospital-level control variables (Core-based statistical area, teaching status, bed size, ownership, system membership, market concentration, annual surgical operations, and inpatient occupancy)
- μᵢ represents hospital-specific random intercepts
- λₜ represents year fixed effects
- δₛ represents state fixed effects
- εᵢₜ represents the error term

All models incorporated hospital-level random intercepts to account for correlation of observations within hospitals over time. Year fixed effects controlled for secular trends affecting all CAHs, while state fixed effects accounted for time-invariant state-level policies and market characteristics. Robust standard errors were clustered at the hospital level to account for serial correlation and heteroskedasticity.

**Data Availability and Transparency**

The NASHP Hospital Cost Tool data are publicly available at <https://tool.nashp.org/>. The AHA Annual Survey data are not available publicly and may be purchased directly from the American Hospital Association.

# Table A1 Effect Estimates of Payer Mix on Profit Margins Among Critical Access Hospitals, 2011-2023

|  | **(1)** | **(2)** | **(3)** | **(4)** |
| --- | --- | --- | --- | --- |
|  | **Operating margin (%)** | **Commercial profit margin (%)** | **Medicare FFS profit margin (%)** | **Medicaid profit margin (%)** |
| Commercial payer mix (%) | - | - | - | - |
| Medicare & MA payer mix (%) | 0.10*** | 0.44*** | 0.01** | -0.22** |
|  | [0.05,0.15] | [0.29,0.59] | [0.00,0.03] | [-0.44,-0.00] |
|  | 0.000 | 0.000 | 0.038 | 0.048 |
| Medicaid & SCHIP & Low-Income payer mix (%) | 0.09*** | 0.97*** | 0.03*** | -0.50*** |
|  | [0.04,0.14] | [0.80,1.15] | [0.01,0.04] | [-0.78,-0.22] |
|  | 0.000 | 0.000 | 0.000 | 0.001 |
| Charity & Uninsured payer mix (%) | -0.18*** | 2.16*** | -0.01 | 0.41* |
|  | [-0.29,-0.07] | [1.84,2.47] | [-0.10,0.08] | [-0.02,0.84] |
|  | 0.001 | 0.000 | 0.793 | 0.062 |
| **CBSA**  **(ref: Rural)** | **-** | **-** | **-** | **-** |
| Metro | 1.46** | 3.95*** | 0.35* | -0.98 |
|  | [0.13,2.79] | [1.33,6.56] | [-0.01,0.71] | [-7.06,5.10] |
|  | 0.032 | 0.003 | 0.059 | 0.753 |
| Micro | 1.07 | 2.27 | 0.18 | -0.22 |
|  | [-0.32,2.46] | [-0.64,5.17] | [-0.12,0.49] | [-5.56,5.12] |
|  | 0.132 | 0.127 | 0.244 | 0.936 |
| **Teaching Status**  **(ref: Non-teaching)** | - | - | - | - |
| Teaching | 1.42*** | 2.86** | 0.30** | 0.43 |
|  | [0.37,2.47] | [0.61,5.10] | [0.04,0.56] | [-6.33,7.18] |
|  | 0.008 | 0.013 | 0.025 | 0.901 |
| **Bed Size**  **(ref: <25)** | - | - | - | - |
| >=25 | 1.57*** | 3.74*** | 0.07 | 2.70 |
|  | [0.59,2.54] | [1.36,6.11] | [-0.19,0.32] | [-1.68,7.07] |
|  | 0.002 | 0.002 | 0.603 | 0.227 |
| **Ownership**  **(ref: Non-profit)** | - | **-** | - | - |
| For-profit | 2.28* | 4.34 | -0.19 | 3.39 |
|  | [-0.36,4.91] | [-2.56,11.24] | [-1.31,0.93] | [-8.29,15.08] |
|  | 0.090 | 0.218 | 0.742 | 0.569 |
| Governmental | -2.32*** | -6.16*** | -0.26** | 1.91 |
|  | [-3.70,-0.94] | [-9.16,-3.17] | [-0.52,-0.00] | [-3.59,7.41] |
|  | 0.001 | 0.000 | 0.049 | 0.496 |
| **System membership (ref: Independent)** | - | **-** | - | - |
| System member | 0.45 | 2.16** | 0.34*** | 1.77 |
|  | [-0.61,1.51] | [0.03,4.28] | [0.09,0.59] | [-2.60,6.14] |
|  | 0.402 | 0.046 | 0.008 | 0.427 |
| **Market Concentration (ref: Unconcentrated (HHI<0.15))** | - | - | - | - |
| Highly concentrated (HHI>=.25) | 0.22 | 0.53 | 0.26* | -0.88 |
|  | [-1.12,1.55] | [-3.15,4.21] | [-0.03,0.54] | [-5.93,4.18] |
|  | 0.752 | 0.778 | 0.080 | 0.734 |
| Moderately concentrated  (HHI: 0.15-<0.25) | -0.68 | -2.25 | 0.15 | -2.95 |
|  | [-1.73,0.37] | [-5.45,0.96] | [-0.10,0.41] | [-7.60,1.69] |
|  | 0.207 | 0.169 | 0.240 | 0.213 |
| **Surgical Operations (ref: Low volume)** | - | - | - | - |
| High volume | 1.68*** | 2.39*** | -0.27** | 5.93*** |
|  | [0.93,2.43] | [0.57,4.22] | [-0.55,-0.00] | [2.33,9.52] |
|  | 0.000 | 0.010 | 0.048 | 0.001 |
| **Inpatient occupancy (%)** | 0.15*** | 0.24*** | -0.00 | 0.28*** |
|  | [0.12,0.18] | [0.18,0.31] | [-0.01,0.01] | [0.18,0.39] |
|  | 0.000 | 0.000 | 0.892 | 0.000 |
| Time fixed effects | Yes | Yes | Yes | Yes |
| State fixed effects | Yes | Yes | Yes | Yes |
| Observations | 15,819 | 15,819 | 15,819 | 15,819 |
| Number of CAHs | 1,384 | 1,384 | 1,384 | 1,384 |

Note: This table presents results from linear mixed-effects models estimating the association between hospital payer mix and four financial outcomes among Critical Access Hospitals from 2011 to 2023. The dependent variables are profit margins by payers, each expressed as a percentage. All models include hospital-level random intercepts (clustered at the hospital level) and fixed effects for year and state. Key independent variables were the percentage of hospital services (by charges) attributable to Commercial, Medicare, Medicaid, and Uncompensated Care payers. Each cell reports the estimated coefficient, followed by the 95% confidence interval in brackets and p-value underneath. Robust standard errors are clustered at the hospital level.

CBSA: Core-based statistical area; HHI: Herfindahl–Hirschman index. MA = Medicare Advantage. SCHIP = State Children’s Health Insurance Program.

***p < 0.01; **p < 0.05; *p < 0.1

# Table A2 Sensitivity Analysis – Winsorization at 1st/99th percentiles

|  | **(1)** | **(2)** | **(3)** | **(4)** |
| --- | --- | --- | --- | --- |
|  | **Operating margin (%)** | **Commercial profit margin (%)** | **Medicare FFS profit margin (%)** | **Medicaid profit margin (%)** |
| Commercial payer mix (%) | - | - | - | - |
| Medicare & MA payer mix (%) | 0.11*** | 0.52*** | 0.02*** | -0.19* |
|  | [0.05,0.16] | [0.38,0.65] | [0.01,0.03] | [-0.40,0.03] |
|  | 0.000 | 0.000 | 0.002 | 0.096 |
| Medicaid & SCHIP & Low-Income payer mix (%) | 0.07*** | 1.03*** | 0.03*** | -0.53*** |
|  | [0.02,0.12] | [0.87,1.19] | [0.02,0.04] | [-0.79,-0.26] |
|  | 0.006 | 0.000 | 0.000 | 0.000 |
| Charity & Uninsured payer mix (%) | -0.22*** | 1.91*** | 0.02** | 0.06 |
|  | [-0.34,-0.11] | [1.55,2.26] | [0.00,0.05] | [-0.39,0.50] |
|  | 0.000 | 0.000 | 0.026 | 0.793 |
| **CBSA**  **(ref: Rural)** | **-** | **-** | **-** | **-** |
| Metro | 1.41* | 4.03*** | 0.19 | -0.58 |
|  | [-0.00,2.83] | [1.30,6.77] | [-0.06,0.45] | [-6.54,5.37] |
|  | 0.050 | 0.004 | 0.137 | 0.848 |
| Micro | 0.96 | 2.31 | 0.14 | 0.39 |
|  | [-0.51,2.43] | [-0.56,5.19] | [-0.10,0.38] | [-4.88,5.67] |
|  | 0.199 | 0.115 | 0.250 | 0.884 |
| **Teaching Status**  **(ref: Non-teaching)** | - | - | - | - |
| Teaching | 1.38** | 1.87* | 0.30*** | 0.31 |
|  | [0.31,2.46] | [-0.26,4.00] | [0.07,0.52] | [-6.00,6.62] |
|  | 0.012 | 0.085 | 0.009 | 0.923 |
| **Bed Size**  **(ref: <25)** | - | - | - | - |
| >=25 | 1.60*** | 3.90*** | 0.02 | 2.86 |
|  | [0.59,2.60] | [1.67,6.14] | [-0.15,0.19] | [-1.56,7.29] |
|  | 0.002 | 0.001 | 0.820 | 0.204 |
| **Ownership**  **(ref: Non-profit)** | - | - | - | - |
| For-profit | 2.65* | 6.33** | 0.49 | 1.64 |
|  | [-0.22,5.53] | [0.44,12.22] | [-0.11,1.10] | [-9.33,12.61] |
|  | 0.071 | 0.035 | 0.111 | 0.770 |
| Governmental | -2.47*** | -6.32*** | -0.40*** | 1.34 |
|  | [-4.02,-0.93] | [-9.16,-3.47] | [-0.60,-0.19] | [-3.80,6.48] |
|  | 0.002 | 0.000 | 0.000 | 0.609 |
| **System membership (ref: Independent)** | - | **-** | - | - |
| System member | 0.46 | 2.13* | 0.26*** | 2.09 |
|  | [-0.69,1.62] | [-0.00,4.26] | [0.09,0.44] | [-2.19,6.36] |
|  | 0.430 | 0.051 | 0.003 | 0.338 |
| **Market Concentration (ref: Unconcentrated (HHI<0.15))** | - | - | - | - |
| Highly concentrated (HHI>=.25) | 0.35 | -1.00 | 0.24* | -1.07 |
|  | [-1.02,1.72] | [-4.16,2.16] | [-0.01,0.49] | [-5.92,3.77] |
|  | 0.614 | 0.535 | 0.056 | 0.664 |
| Moderately concentrated  (HHI: 0.15-<0.25) | -0.69 | -2.17 | 0.03 | -3.02 |
|  | [-1.76,0.39] | [-4.81,0.47] | [-0.20,0.25] | [-7.41,1.38] |
|  | 0.210 | 0.107 | 0.823 | 0.178 |
| **Surgical Operations (ref: Low volume)** | - | - | - | - |
| High volume | 1.74*** | 2.28*** | -0.09 | 6.24*** |
|  | [0.94,2.55] | [0.56,4.01] | [-0.28,0.09] | [2.87,9.60] |
|  | 0.000 | 0.010 | 0.326 | 0.000 |
| **Inpatient occupancy (%)** | 0.15*** | 0.24*** | -0.00 | 0.27*** |
|  | [0.12,0.18] | [0.18,0.30] | [-0.01,0.00] | [0.17,0.37] |
|  | 0.000 | 0.000 | 0.429 | 0.000 |
| Time fixed effects | Yes | Yes | Yes | Yes |
| State fixed effects | Yes | Yes | Yes | Yes |
| Observations | 16,141 | 16,141 | 16,141 | 16,141 |
| Number of CAHs | 1,387 | 1,387 | 1,387 | 1,387 |

Note: This table presents sensitivity analyses using profit margin variables winsorized at the 1st and 99th percentiles. The dependent variables are winsorized profit margins by payers, each expressed as a percentage. All models are linear mixed-effects models with hospital-level random intercepts and fixed effects for year and state. Key independent variables were the percentage of hospital services (by charges) attributable to Commercial, Medicare, Medicaid, and Uncompensated Care payers. Control variables include CBSA category, teaching status, bed size, ownership type, health system membership, market concentration (HHI), surgical volume category, and inpatient occupancy. Each cell reports the estimated coefficient, followed by the 95% confidence interval in brackets and p-value underneath. Robust standard errors are clustered at the hospital level.

CBSA: Core-based statistical area; HHI: Herfindahl–Hirschman index. MA = Medicare Advantage. SCHIP = State Children’s Health Insurance Program.

***p < 0.01; **p < 0.05; *p < 0.1

# Table A3 Sensitivity Analysis – Balanced Panel

|  | **(1)** | **(2)** | **(3)** | **(4)** |
| --- | --- | --- | --- | --- |
|  | **Operating margin (%)** | **Commercial profit margin (%)** | **Medicare FFS profit margin (%)** | **Medicaid profit margin (%)** |
| Commercial payer mix (%) | - | - | - | - |
| Medicare & MA payer mix (%) | 0.11*** | 0.46*** | 0.01* | -0.11 |
|  | [0.05,0.17] | [0.32,0.60] | [-0.00,0.03] | [-0.36,0.15] |
|  | 0.000 | 0.000 | 0.051 | 0.408 |
| Medicaid & SCHIP & Low-Income payer mix (%) | 0.08*** | 0.84*** | 0.04*** | -0.25 |
|  | [0.03,0.14] | [0.63,1.04] | [0.02,0.06] | [-0.59,0.10] |
|  | 0.005 | 0.000 | 0.000 | 0.167 |
| Charity & Uninsured payer mix (%) | -0.24*** | 1.92*** | -0.08 | 0.09 |
|  | [-0.38,-0.10] | [1.55,2.28] | [-0.25,0.09] | [-0.57,0.75] |
|  | 0.001 | 0.000 | 0.360 | 0.778 |
| **CBSA**  **(ref: Rural)** | **-** | **-** | **-** | **-** |
| Metro | 1.53** | 2.40* | 0.30 | 3.54 |
|  | [0.04,3.02] | [-0.21,5.01] | [-0.19,0.80] | [-2.68,9.77] |
|  | 0.044 | 0.071 | 0.228 | 0.265 |
| Micro | 0.94 | 1.56 | 0.32 | -2.01 |
|  | [-0.56,2.45] | [-1.32,4.43] | [-0.09,0.74] | [-8.54,4.52] |
|  | 0.219 | 0.289 | 0.126 | 0.547 |
| **Teaching Status**  **(ref: Non-teaching)** | - | - | - | - |
| Teaching | 1.51** | 2.74** | 0.25 | 1.53 |
|  | [0.27,2.75] | [0.42,5.06] | [-0.09,0.60] | [-5.60,8.66] |
|  | 0.017 | 0.020 | 0.152 | 0.674 |
| **Bed Size**  **(ref: <25)** | - | - | - | - |
| >=25 | 1.29** | 2.91** | 0.06 | 2.07 |
|  | [0.25,2.34] | [0.44,5.37] | [-0.27,0.40] | [-3.05,7.19] |
|  | 0.015 | 0.021 | 0.720 | 0.428 |
| **Ownership**  **(ref: Non-profit)** | - | **-** | - | - |
| For-profit | 0.51 | 5.76 | -1.15 | -3.04 |
|  | [-2.94,3.97] | [-2.00,13.52] | [-3.70,1.40] | [-18.47,12.38] |
|  | 0.771 | 0.146 | 0.377 | 0.699 |
| Governmental | -1.84** | -6.93*** | -0.14 | 1.14 |
|  | [-3.26,-0.42] | [-10.29,-3.57] | [-0.48,0.20] | [-5.15,7.43] |
|  | 0.011 | 0.000 | 0.425 | 0.722 |
| **System membership (ref: Independent)** | - | **-** | - | - |
| System member | 0.22 | -0.13 | 0.36* | 2.21 |
|  | [-1.01,1.46] | [-2.40,2.14] | [-0.02,0.75] | [-2.86,7.27] |
|  | 0.725 | 0.912 | 0.065 | 0.393 |
| **Market Concentration (ref: Unconcentrated (HHI<0.15))** | - | - | - | - |
| Highly concentrated (HHI>=.25) | 0.84 | 0.39 | 0.36** | 0.84 |
|  | [-0.58,2.26] | [-3.10,3.87] | [0.03,0.69] | [-4.74,6.42] |
|  | 0.245 | 0.828 | 0.032 | 0.768 |
| Moderately concentrated  (HHI: 0.15-<0.25) | 0.01 | -0.85 | 0.13 | 0.42 |
|  | [-1.01,1.03] | [-3.55,1.86] | [-0.20,0.45] | [-4.75,5.59] |
|  | 0.984 | 0.540 | 0.436 | 0.873 |
| **Surgical Operations (ref: Low volume)** | - | - | - | - |
| High volume | 1.53*** | 3.43*** | -0.36** | 4.93** |
|  | [0.57,2.49] | [1.52,5.35] | [-0.71,-0.01] | [0.39,9.48] |
|  | 0.002 | 0.000 | 0.042 | 0.033 |
| **Inpatient occupancy (%)** | 0.16*** | 0.23*** | 0.00 | 0.29*** |
|  | [0.13,0.19] | [0.16,0.29] | [-0.00,0.01] | [0.17,0.42] |
|  | 0.000 | 0.000 | 0.361 | 0.000 |
| Time fixed effects | Yes | Yes | Yes | Yes |
| State fixed effects | Yes | Yes | Yes | Yes |
| Observations | 9,971 | 9,971 | 9,971 | 9,971 |
| Number of CAHs | 767 | 767 | 767 | 767 |

Note: This table presents sensitivity analyses restricting the sample to a balanced panel (i.e., Critical Access Hospitals observed in the 13 study years). The dependent variables are profit margins by payers, each expressed as a percentage. All models are linear mixed-effects models with hospital-level random intercepts and fixed effects for year and state. Key independent variables were the percentage of hospital services (by charges) attributable to Commercial, Medicare, Medicaid, and Uncompensated Care payers. Control variables include CBSA category, teaching status, bed size, ownership type, health system membership, market concentration (HHI), surgical volume category, and inpatient occupancy. Each cell reports the estimated coefficient, followed by the 95% confidence interval in brackets and p-value underneath. Robust standard errors are clustered at the hospital level.

CBSA: Core-based statistical area; HHI: Herfindahl–Hirschman index. MA = Medicare Advantage. SCHIP = State Children’s Health Insurance Program.

***p < 0.01; **p < 0.05; *p < 0.1

# Table A4 Sensitivity Analysis – Alternative Models with Medicaid Expansion Control

|  | **(1)** | **(2)** | **(3)** | **(4)** |
| --- | --- | --- | --- | --- |
|  | **Operating margin (%)** | **Commercial profit margin (%)** | **Medicare FFS profit margin (%)** | **Medicaid profit margin (%)** |
| Commercial payer mix (%) | - | - | - | - |
| Medicare & MA payer mix (%) | 0.10*** | 0.44*** | 0.01** | -0.22** |
|  | [0.05,0.15] | [0.29,0.58] | [0.00,0.03] | [-0.44,-0.00] |
|  | 0.000 | 0.000 | 0.037 | 0.045 |
| Medicaid & SCHIP & Low-Income payer mix (%) | 0.09*** | 0.97*** | 0.03*** | -0.52*** |
|  | [0.04,0.14] | [0.80,1.15] | [0.01,0.04] | [-0.80,-0.23] |
|  | 0.000 | 0.000 | 0.000 | 0.000 |
| Charity & Uninsured payer mix (%) | -0.19*** | 2.16*** | -0.01 | 0.45** |
|  | [-0.30,-0.07] | [1.83,2.48] | [-0.10,0.08] | [0.01,0.88] |
|  | 0.001 | 0.000 | 0.796 | 0.043 |
| Medicaid Expansion (ref: Non-Medicaid Expansion) | - | - | - | - |
|  | -0.13 | 0.07 | 0.02 | 2.27 |
|  | [-0.93,0.67] | [-2.51,2.65] | [-0.28,0.31] | [-2.26,6.80] |
|  | 0.748 | 0.960 | 0.911 | 0.326 |
| **CBSA**  **(ref: Rural)** | **-** | **-** | **-** | **-** |
| Metro | 1.46** | 3.94*** | 0.35* | -1.03 |
|  | [0.13,2.79] | [1.32,6.57] | [-0.01,0.71] | [-7.13,5.06] |
|  | 0.031 | 0.003 | 0.060 | 0.740 |
| Micro | 1.06 | 2.27 | 0.18 | -0.19 |
|  | [-0.32,2.45] | [-0.64,5.17] | [-0.12,0.49] | [-5.54,5.15] |
|  | 0.133 | 0.126 | 0.244 | 0.944 |
| **Teaching Status**  **(ref: Non-teaching)** | - | - | - | - |
| Teaching | 1.42*** | 2.75** | 0.30** | 0.61 |
|  | [0.38,2.46] | [0.54,4.95] | [0.04,0.57] | [-6.16,7.37] |
|  | 0.008 | 0.015 | 0.022 | 0.860 |
| **Bed Size**  **(ref: <25)** | - | - | - | - |
| >=25 | 1.42*** | 2.86** | 0.30** | 0.40 |
|  | [0.38,2.47] | [0.62,5.10] | [0.04,0.56] | [-6.35,7.16] |
|  | 0.008 | 0.012 | 0.025 | 0.907 |
| **Ownership**  **(ref: Non-profit)** | - | **-** | - | - |
| For-profit | 2.27* | 4.34 | -0.19 | 3.43 |
|  | [-0.36,4.91] | [-2.56,11.24] | [-1.31,0.93] | [-8.26,15.11] |
|  | 0.091 | 0.217 | 0.742 | 0.566 |
| Governmental | -2.33*** | -6.16*** | -0.26** | 1.99 |
|  | [-3.71,-0.94] | [-9.15,-3.18] | [-0.52,-0.00] | [-3.50,7.48] |
|  | 0.001 | 0.000 | 0.050 | 0.478 |
| **System membership (ref: Independent)** | - | **-** | - | - |
| System member | 0.45 | 2.16** | 0.34*** | 1.82 |
|  | [-0.61,1.51] | [0.03,4.28] | [0.09,0.59] | [-2.55,6.19] |
|  | 0.406 | 0.046 | 0.008 | 0.415 |
| **Market Concentration (ref: Unconcentrated (HHI<0.15))** | - | - | - | - |
| Highly concentrated (HHI>=.25) | 0.22 | 0.53 | 0.26* | -0.93 |
|  | [-1.12,1.55] | [-3.15,4.20] | [-0.03,0.54] | [-5.99,4.14] |
|  | 0.748 | 0.778 | 0.081 | 0.720 |
| Moderately concentrated  (HHI: 0.15-<0.25) | -0.68 | -2.25 | 0.15 | -2.91 |
|  | [-1.73,0.37] | [-5.46,0.96] | [-0.10,0.41] | [-7.55,1.73] |
|  | 0.205 | 0.170 | 0.239 | 0.219 |
| **Surgical Operations (ref: Low volume)** | - | - | - | - |
| High volume | 1.68*** | 2.39** | -0.27** | 5.91*** |
|  | [0.93,2.43] | [0.57,4.22] | [-0.55,-0.00] | [2.32,9.51] |
|  | 0.000 | 0.010 | 0.048 | 0.001 |
| **Inpatient occupancy (%)** | 0.15*** | 0.24*** | -0.00 | 0.29*** |
|  | [0.12,0.18] | [0.18,0.31] | [-0.01,0.01] | [0.18,0.39] |
|  | 0.000 | 0.000 | 0.894 | 0.000 |
| Time fixed effects | Yes | Yes | Yes | Yes |
| State fixed effects | Yes | Yes | Yes | Yes |
| Observations | 15,819 | 15,819 | 15,819 | 15,819 |
| Number of CAHs | 1,384 | 1,384 | 1,384 | 1,384 |

Note: This table presents results from linear mixed-effects models adding a Medicaid expansion indicator as a covariate. The Medicaid expansion variable equals 1 for hospital-year observations in states that had implemented Medicaid expansion under the Affordable Care Act by that year, and 0 otherwise. Expansion timing was coded based on each state's implementation date using data from the Kaiser Family Foundation. The dependent variables are profit margins by payers, each expressed as a percentage. All models include hospital-level random intercepts and fixed effects for year and state. Key independent variables were the percentage of hospital services (by charges) attributable to Commercial, Medicare, Medicaid, and Uncompensated Care payers. Each cell reports the estimated coefficient, followed by the 95% confidence interval in brackets and p-value underneath. Robust standard errors are clustered at the hospital level. CBSA: Core-based statistical area; HHI: Herfindahl–Hirschman index. MA = Medicare Advantage. SCHIP = State Children’s Health Insurance Program. ***p < 0.01; **p < 0.05; *p < 0.1
